# Craniopharyngioma presenting with severe hyponatremia, hyponatremia-induced myopathy, and panhypopituitarism: a case report

**DOI:** 10.1186/s13256-017-1210-x

**Published:** 2017-02-05

**Authors:** M. D. S. A. Dilrukshi, G. V. N. Sandakumari, P. K. Abeysundara, T. Chang

**Affiliations:** 1 University Medical Unit, National Hospital of Sri Lanka, Colombo 10, Sri Lanka; 2 Department of Clinical Medicine, University of Colombo, Colombo, Sri Lanka

**Keywords:** Craniopharyngioma, Hyponatremia, Myopathy, Panhypopituitarism

## Abstract

**Background:**

Craniopharyngiomas are rare intracranial tumors commonly presenting with neurological symptoms. Reports of severe hyponatremia as a presenting manifestation of a craniopharyngioma and hyponatremia-induced myopathy are rare. We report the case of a patient with craniopharyngioma presenting with severe hyponatremia, panhypopituitarism, and hyponatremia-induced myopathy.

**Case presentation:**

A 52-year-old Sri Lankan man presented with anorexia, nausea, fatigue, generalized muscle weakness, and cramps for 1 week. The onset of his illness had been preceded by vomiting and diarrhea for 1 day which he attributed to food poisoning. On examination, he had an apathetic disposition with a generalized “sallow complexion.” He was not dehydrated. Apart from reduced muscle power (4/5) and hyporeflexia, the neurological examination was normal. His serum sodium was 102 mmol/l; potassium 4.1 mmol/l; chloride 63 mmol/l; plasma osmolality 272 mosm/KgH_2_O; urine osmolality 642 mosm/KgH_2_O; and urine sodium 79 mmol/l. His creatine phosphokinase was 12,400 U/l, lactate dehydrogenase 628 U/l, aspartate aminotransferase 360 U/l, and alanine aminotransferase 64 U/l. His hormone profile revealed panhypopituitarism. An electromyogram showed nonspecific abnormalities while a muscle biopsy did not show any pathology. Magnetic resonance imaging of his brain demonstrated a well-defined craniopharyngioma with suprasellar extension. His pituitary gland was compressed and the pituitary stalk was displaced by the tumor. He had marked improvement in muscle power and rapid reduction of serum creatine phosphokinase levels paralleling the correction of severe hyponatremia, even before the initiation of hormone replacement.

**Conclusions:**

This case illustrates the rare presentation of severe hyponatremia and hyponatremia-induced myopathy in patients with craniopharyngioma, awareness of which would facilitate early appropriate investigations and treatment.

## Background

Craniopharyngiomas are rare, solid, or solid-mixed tumors that arise from remnants of the Rathke pouch along a line from the nasopharynx to the diencephalon [1]. They are slow growing tumors which clinically manifest only after the tumor grows to a size of around 3 cm or more. The majority of patients (75 %) have been reported to present with neurological symptoms [2], with headache being the commonest (82 %). Symptoms of endocrine dysfunction are also common (66 to 90 %) presenting manifestations [2], but symptomatic severe hyponatremia as a presenting symptom is rare [3–5]. Reports of hyponatremia-induced myopathy, which is a recognized non-neurological manifestation of severe hyponatremia caused by skeletal muscle disruption, are sparse [6–9]. We report the case of a patient with a craniopharyngioma presenting with severe hyponatremia, panhypopituitarism, and hyponatremia-induced myopathy.

## Case presentation

A 52-year-old previously healthy Sri Lankan man presented with anorexia, nausea, fatigue, generalized muscle weakness, and cramps for 1 week. The onset of his illness had been preceded by a day of vomiting and diarrhea which he attributed to food poisoning. On admission he had constipation, moderate frontal headache, and generalized body weakness but did not report abdominal pain, fever, cough, or history of polyuria and increased thirst.

On examination, he had an apathetic disposition with a generalized “sallow complexion.” He was not dehydrated. Apart from reduced muscle power (4/5) and hyporeflexia, the neurological examination was normal. His pulse rate was 68 beats per minute and his blood pressure was 120/80 mmHg. The rest of the physical examination was normal.

His serum sodium was 102 mmol/l (135 to 145 mmol/l); potassium 4.1 mmol/l (3.5 to 5 mmol/l); chloride 63 mmol/l (98 to 108 mmol/l); plasma osmolality 272 mosm/KgH_2_O (275 to 295 mosm/KgH2O); urine osmolality 642 mosm/KgH_2_O (300 to 900 mosm/KgH2O); and urine sodium 79 mmol/l (40 to 220 mmol/l). His serum calcium was 7.7 mg/dl (normal 8.5 to 10.5 mg/dl); phosphate 2.6 mmol/l (0.8 to 1.5 mmol/l); creatine kinase 12,400 U/l (normal up to 25 to 174 U/l); lactate dehydrogenase (LDH) 628 U/l (normal up to 140 to 280 U/l); aspartate aminotransferase (AST) 360 U/l (normal up to 10 to 35 U/l); and alanine aminotransferase (ALT) 64 U/l (normal up to 10 to 40 U/l). His hormone profile revealed a serum prolactin of 234 mU/l (normal up to 340 mU/l); luteinizing hormone (LH) 0.6 U/L (normal 1 to 10 U/L); follicle-stimulating hormone (FSH) 1.1 U/l (normal 1 to 7 U/L); total testosterone 31.1 ng/dl (normal 241 to 827 ng/dl); thyroid-stimulating hormone (TSH) 0.62 mIU/l (normal 0.55 to 4.7 mIU/l); free thyroid hormone (FT_4_) 0.46 ng/dl (normal 0.89 to 1.76 ng/dl), 9 a.m. serum cortisol 69.18 nmol/l (normal 118.6 to 618 nmol/l), and an inadequate response to the short Synacthen test.

**Fig. 1:**
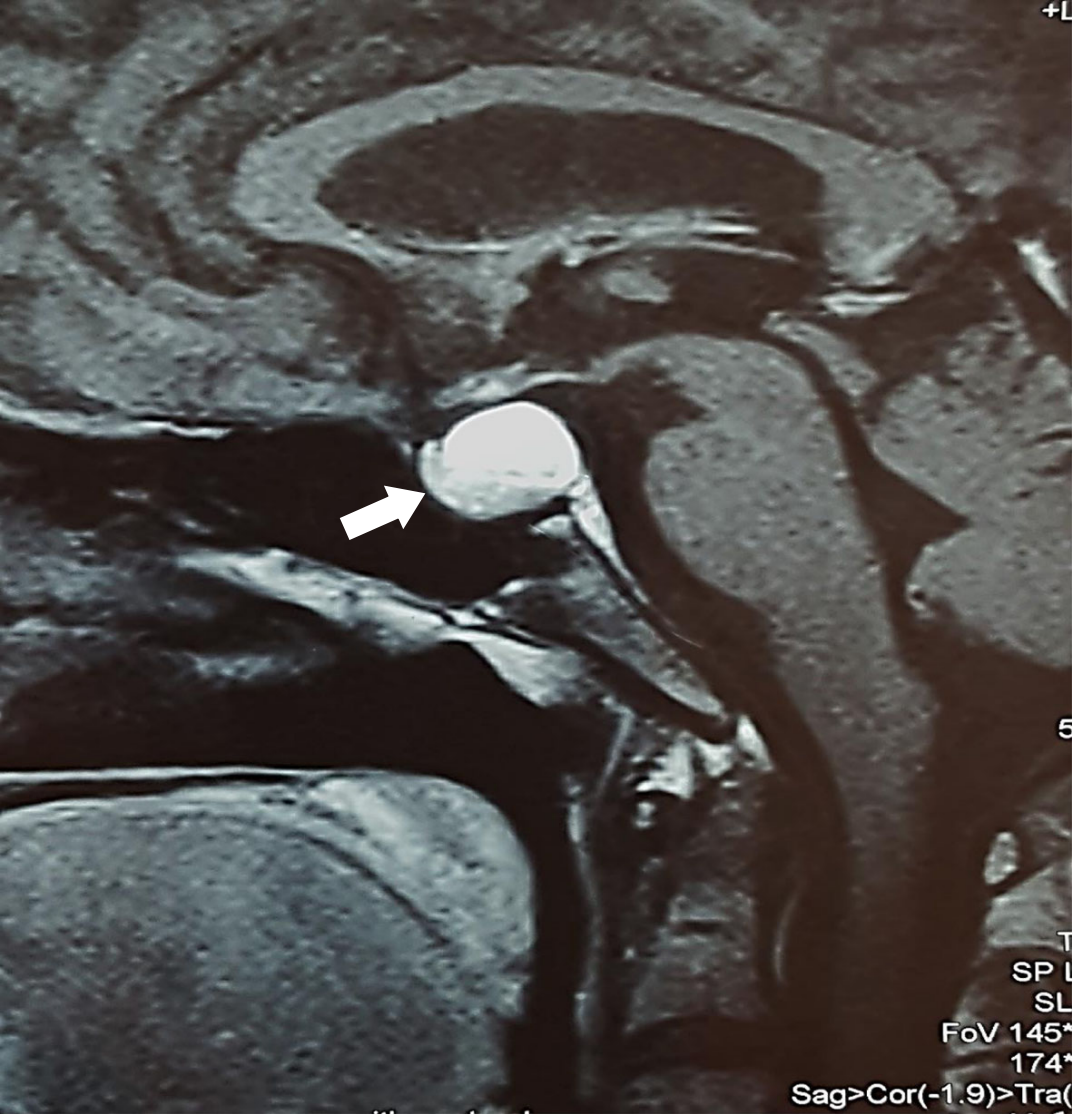
T1-weighted contrast-enhanced magnetic resonance imaging of the brain demonstrates a well-defined craniopharyngioma (*arrow*) within the pituitary fossa with suprasellar extension. The pituitary gland is compressed and the pituitary stalk is displaced by the tumor

His hemoglobin concentration, leukocyte count, and platelet count were within normal limits. His erythrocyte sedimentation rate was 30 mm/hour while his C-reactive protein was 8.2 mg/l (normal up to 6). His urine analysis was normal. An ultrasound scan of his abdomen and a chest radiograph were normal. An electromyogram showed nonspecific abnormalities while a muscle biopsy did not show any pathology. Magnetic resonance imaging of his brain demonstrated a well-defined craniopharyngioma within his pituitary fossa measuring 1.3×1.6×1.6 cm with evidence of suprasellar extension (Fig. 1). His pituitary gland was compressed and the pituitary stalk was displaced by the tumor.

He was treated with 3 % saline during the acute phase, which led to a marked improvement in his muscle power and rapid reduction of his serum creatine phosphokinase (CPK) levels, which paralleled the correction of severe hyponatremia over 2 to 3 days. Despite his euvolemic hyponatremic state, we did not restrict his fluid intake because of the risk of rhabdomyolysis-induced acute kidney injury given that he had markedly elevated serum CPK. Furthermore, the diagnosis of panhypopituitarism was delayed because this possibility was not initially suspected and was compounded by the delay in obtaining confirmatory hormonal tests which are not routinely done in our hospital. Once the diagnosis of panhypopituitarism was established, hormone replacement therapy was commenced with oral hydrocortisone followed by thyroxine after an interval of 1 week.

## Discussion

Our patient had an inadequate TSH increment in response to very low FT_4_ levels (suggestive of secondary hypothyroidism) associated with very low levels of FSH, LH, and testosterone levels (suggestive of secondary hypogonadism). In the context of absence of hyperpigmentation typically seen in primary hypoadrenalism due to elevated serum adrenocorticotropic hormone (ACTH) levels and absence of severe hyperkalemia typical of mineralocorticoid deficiency, his inadequate response to the short Synacthen test was considered to be due to secondary hypoadrenalism. The panhypopituitarism state in our patient occurred due to the compression of his pituitary gland by the tumor.

Severe hyponatremia is defined as a serum sodium level <120 mmol/L, which may lead to fatal neurological and non-neurological complications [10]. Our patient had hypotonic hyponatremia, with high urine osmolality and high urine sodium excretion. He was euvolemic and there was no clinical or laboratory evidence of renal, liver, or heart failure nor history suggestive of diuretic abuse. Hypopituitarism was thought to cause hyponatremia in our patient. Although hyponatremia is known to occur in patients with hypopituitarism, severe hyponatremia of the degree seen in our patient occurring as a presenting feature of hypopituitarism is rare [11], indicating an additional contributory factor. Local tumors in the region of the pituitary, such as craniopharyngiomas, can cause mechanical compression of the pituitary stalk leading to an excessive or inappropriate secretion of antidiuretic hormone (SIADH) due to interruption of the inhibitory system. There are few reports of patients with SIADH with pituitary tumors and normal pituitary functions, where local mechanical stress is a plausible cause of SIADH [12]. SIADH can cause severe hyponatremia. However, other possible causes of SIADH, such as other malignancies elsewhere, pulmonary disease, drugs, and central nervous system infections were excluded by relevant history, laboratory investigations, and relevant imaging studies. Since adrenal insufficiency and hypothyroidism biochemically can mimic SIADH [13], early accurate diagnosis of a tumor in the pituitary region or a craniopharyngioma depends upon a high level of clinical suspicion of a suprasellar lesion and early brain imaging.

Thus far, there have been only few cases of hyponatremia-induced myopathy reported [6–9] and no report describes its occurrence in the presence of a craniopharyngioma. Our patient who was diagnosed as having a craniopharyngioma presented with generalized muscle weakness and elevated muscle enzymes (CPK, LDH, and AST) associated with severe hyponatremia, which was suggestive of rhabdomyolysis. His low serum calcium and high serum phosphate levels were consistent with rhabdomyolysis and, therefore, an alternative cause such as vitamin D deficiency was not considered. His elevated serum AST was also thought to be due to rhabdomyolysis rather than coming from the liver since it gradually normalized with the correction of hyponatremia. Furthermore, there was no clinical or radiological evidence of liver injury. Other causes of myopathies such as inflammatory, paraneoplastic, infectious, drugs induced, and toxin induced, and other metabolic causes were excluded in our patient by detailed history, examination, biochemical investigations, and histopathological investigations relevant to the clinical context. A marked improvement in his muscle power and rapid reduction of serum CPK levels paralleling the correction of severe hyponatremia, even before initiating steroid and other hormone replacement, supports the diagnosis of hyponatremia-induced myopathy in our patient.

There is no established hypothesis to explain the pathophysiology of hyponatremia-induced myopathy. One theory is that a reduced extracellular sodium level may disturb the cell membrane sodium-calcium pumps, which leads to increased intracellular calcium and activation of cellular protease and lipase causing rhabdomyolysis. Another theory is that in acute hyponatremia, due to lower osmolality of extracellular fluid, cells start to swell and in order to maintain cell membrane integrity, intracellular potassium is extruded for hours, which in turn lowers the transmembrane potential. If the hyponatremia state is not corrected, the stressed cells release creatine kinase and myoglobin with cell injury [6, 7, 14]. The serum creatine kinase may elevate after 48 to 96 hours of hyponatremia either due to the lowered sodium level itself or the rapid correction of hyponatremia [15].

## Conclusions

Severe hyponatremia can be a rare presenting feature of a craniopharyngioma. A high level of clinical suspicion and early brain imaging may be helpful in managing patients with severe hyponatremia accurately. Myopathy is a rare complication of severe hyponatremia, which warrants early recognition and treatment to prevent rhabdomyolysis and acute kidney injury.

## Abbreviations

ACTH: Adrenocorticotropic hormone
ALT: Alanine aminotransferase
AST: Aspartate aminotransferase
CPK: Creatine phosphokinase
FSH: Follicle-stimulating hormone
FT_4_: Free thyroid hormone
LDH: Lactate dehydrogenase
LH: Luteinizing hormone
SIADH: Syndrome of inappropriate secretion of antidiuretic hormone
TSH: Thyroid-stimulating hormone
